# Suppression of Neurotoxic Lesion-Induced Seizure Activity: Evidence for a Permanent Role for the Hippocampus in Contextual Memory

**DOI:** 10.1371/journal.pone.0027426

**Published:** 2011-11-14

**Authors:** Fraser T. Sparks, Hugo Lehmann, Khadaryna Hernandez, Robert J. Sutherland

**Affiliations:** 1 Department of Neuroscience, Canadian Centre for Behavioural Neuroscience, The University of Lethbridge, Lethbridge, Alberta, Canada; 2 Department of Psychology, Trent University, Peterborough, Ontario, Canada; Université Pierre et Marie Curie, France

## Abstract

Damage to the hippocampus (HPC) using the excitotoxin N-methyl-D-aspartate (NMDA) can cause retrograde amnesia for contextual fear memory. This amnesia is typically attributed to loss of cells in the HPC. However, NMDA is also known to cause intense neuronal discharge (seizure activity) during the hours that follow its injection. These seizures may have detrimental effects on retrieval of memories. Here we evaluate the possibility that retrograde amnesia is due to NMDA-induced seizure activity or cell damage *per se*. To assess the effects of NMDA induced activity on contextual memory, we developed a lesion technique that utilizes the neurotoxic effects of NMDA while at the same time suppressing possible associated seizure activity. NMDA and tetrodotoxin (TTX), a sodium channel blocker, are simultaneously infused into the rat HPC, resulting in extensive bilateral damage to the HPC. TTX, co-infused with NMDA, suppresses propagation of seizure activity. Rats received pairings of a novel context with foot shock, after which they received NMDA-induced, TTX+NMDA-induced, or no damage to the HPC at a recent (24 hours) or remote (5 weeks) time point. After recovery, the rats were placed into the shock context and freezing was scored as an index of fear memory. Rats with an intact HPC exhibited robust memory for the aversive context at both time points, whereas rats that received NMDA or NMDA+TTX lesions showed a significant reduction in learned fear of equal magnitude at both the recent and remote time points. Therefore, it is unlikely that observed retrograde amnesia in contextual fear conditioning are due to disruption of non-HPC networks by propagated seizure activity. Moreover, the memory deficit observed at both time points offers additional evidence supporting the proposition that the HPC has a continuing role in maintaining contextual memories.

## Introduction

In experiments designed to assess the role of the HPC in memory in rats, HPC damage produced by selective infusions of the neurotoxin N-methyl-D-aspartate (NMDA) have produced robust amnesia [1]–[4]. NMDA mediated HPC damage is based upon the excitotoxic effects of glutamate receptor agonism, and has proven effective in ablating the principal subfields, hilar cells, and dentate gyrus [5]. Though the damage produced by neurotoxic lesions has a high degree of selectivity, lesions of the HPC can cause less obvious disruption to distal network circuitry [5], [6]. This has been a concern using neurotoxic lesions, as well as other permanent lesion techniques [7]–[9]. Infusions of NMDA can produce HPC focused seizure activity that propagates to distal networks [10]. Because the seizure activity can be severe, it is important to determine the extent to which retrograde amnesia found after HPC lesions [1], [11] is influenced by disruptive seizure activity propagating to non-HPC regions. This seizure activity may not be as great a concern when using non-neurotoxic lesion techniques, therefore leaving open the possibility that seizure activity could mediate differences in experimental results.

Retrograde amnesia for contextual fear memory is observed following damage to the hippocampus (HPC) [1]–[4], [12]–[14]. To assess the possibility that lesion-induced seizure activity contributes to retrograde amnesia for contextual fear memory, we employed a novel lesion technique that enabled control of seizure severity. This technique utilized the neurotoxic effects of NMDA and concurrently reduced HPC focused synchronous activity by blocking sodium channel conduction using tetrodotoxin (TTX). We damaged the HPC using the standard NMDA or the reduced seizure lesion technique at multiple time points following learning. Rats learned to fear a context during a single conditioning session, and extensive HPC lesions were produced following training.

Studies using lesions to investigate if contextual fear memory dependency on the HPC changes with the passage of time, have produced mixed results (see [4], [15] for in-depth review). The effects of HPC damage in contextual fear memory tasks typically do not differentially affect memories of different ages, meaning that recently acquired memories are just as susceptible to HPC damage as remote memories acquired long before the damage [1]–[4]. Though equivalent retrograde amnesia for recent and remote context fear memory is demonstrated in these studies, there are some accounts suggesting that remote context fear memory survives damage to the HPC, a pattern of results termed *temporally graded retrograde amnesia* [12]–[14], [16], [17]. Sparing of remote memories after HPC damage suggests that the HPC is no longer necessary for recall of these memories, and is taken as support for the idea of temporally based systems consolidation processes [7], [18]–[23]. Strong evidence for a systems consolidation process is not found within recent rodent experiments [4], [15]. Therefore it is necessary to determine why a few studies do show temporally graded retrograde amnesia [12]–[14]. To test the possibility that neurotoxic lesion induced seizure activity is responsible for the discrepant results, we compared high and low seizure inducing lesions at multiple training-to-surgery intervals (1 week and 5 weeks). Thus our results include retention performance for recent and remote context fear memories. The dependence of context fear memory on the HPC is shown following NMDA mediated damage while modulating the amount of seizure activity.

## Results

### NMDA produces pronounced tissue damage

**Figure 1:**
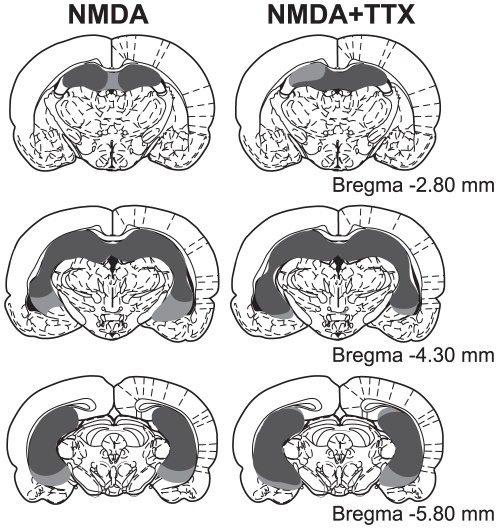
Histology. Illustration of the smallest (dark grey) and largest (light grey) lesion observed bilaterally through the rostral and caudal extent of the HPC for each lesion group.

**Figure 2:**
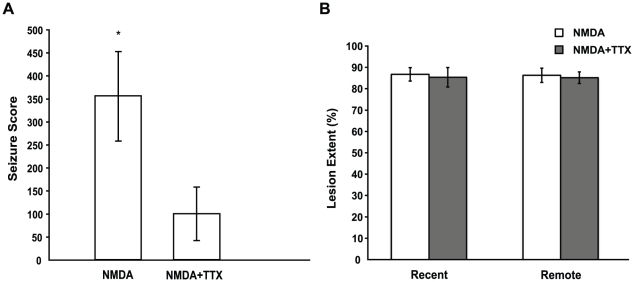
Post-operative seizure scores and HPC damage. (A) Mean ± SEM seizure rating scores calculated for the 3 hr post-operative period, using equation (2). TTX significantly decreased NMDA mediated seizure activity (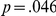). (B) Mean ± SEM lesion extent calculated for each lesion group at the Recent and Remote intervals. All lesion groups sustained equivalent HPC damage.

The NMDA injections produced extensive cell loss in all of the principal subfields (CA1-CA3) of the HPC, the dentate gyrus, as well as the anterior portion of the subiculum for each rat in both the NMDA alone and NMDA+TTX groups at the recent and remote time points. Figure 1 shows a schematic reconstruction of the extent of brain damage caused by the NMDA infusions. Using unbiased stereology [24], it is estimated that 86.0% of the HPC was damaged by infusions of NMDA alone (SD = 6.7; Min = 74.5; Max = 93.7), and 84.8% of the HPC was damaged by the combined infusion of NMDA and TTX (SD = 5.5; Min = 77.7; Max = 93.8). Rats estimated to have less than 70% total damage were removed from analysis, yielding NMDA group n = 13 and NMDA+TTX n = 14 (see Figure 2B). The amount of HPC damage did not differ between these two lesion conditions (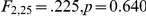) at either interval (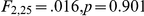). Damage to the HPC extended throughout the dorsal and ventral HPC. The lowering and withdrawal of the injection cannulae also caused minor damage in the parietal cortex. No noticeable damage was found in the thalamus or basolateral and central region of the amygdala in any of the rats. Note that the lesion size estimates did not include one rat from the NMDA and three from the NMDA+TTX groups because of difficulties with the histology method, but the behavioural scores of these rats were retained and used in the behavioural analysis.

### TTX controls seizure severity

NMDA induced seizures were assessed for the first 3 hours following surgery. The rating scale that was used ranks bouts of seizure activity by severity and was modified from that previously used in kindling studies [25], [26]. The following scores were used for rating severity of NMDA induced seizures: 0, no seizures; 1, eye closure, twitching of vibrissae, sniffling, facial clonus, staring; 2, head nodding associated with more severe facial clonus; 3, unilateral or bilateral forelimb clonus; 4, rearing, often accompanied by bilateral forelimb clonus; 5, rearing with loss of balance and falling accompanied by generalized clonic seizures; 6, sustained generalized clonic convulsions (convulsive status epilepticus); 7, jumping/tonic seizure; and 8, respiratory arrest. A total seizure score was calculated for each rat by first taking the sum of the seizure durations for each rating, multiplying this sum by the rating number, and finally taking the sum of the rating x duration products. This seizure score allows for comparison of the duration and severity of seizures across groups (see Figure 2A). Details of the seizure score equation (1) are as follows: *S* is the total seizure score for an individual rat, *r* is the rating number, *d* is the individual seizure durations for each rating *r*, and *n* is the number of seizures for each rating number. 
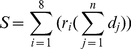
(1)


The rats of the sham group underwent the same scoring procedure, and none showed signs of seizure activity, therefore they were not included in the analysis. In addition, two rats from the NMDA+TTX group had incomplete video data, so were excluded from the analysis. An independent-samples *t*-test was conducted to compare seizure scores for the NMDA and NMDA+TTX lesion groups, revealing a significant difference (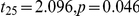) between the two groups. Moreover, the seizure scores for the NMDA+TTX lesion group were not significantly greater than the sham value of zero (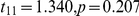). Taken together, these results indicate that infusion of NMDA produced significant levels of seizure activity, and that TTX was effective at suppressing this NMDA mediated seizure activity to a level indistinguishable from controls. Furthermore this degree of seizure reduction did not affect the extent of NMDA-induced HPC damage.

### HPC damage results in retrograde amnesia for both recent and remote memories

**Figure 3:**
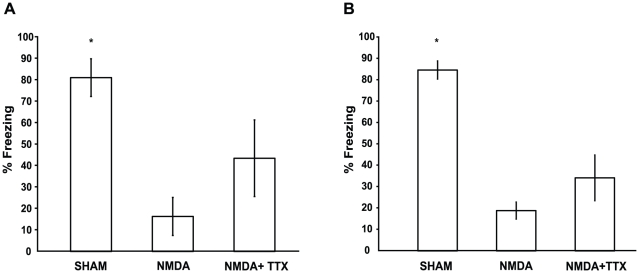
Fear conditioning retention sessions. Mean ± SEM (A) Recent time point. Percentage freezing during the context memory retention session for rats that underwent contextual fear conditioning 1 week prior to HPC surgery. Sham rats froze significantly more than either lesion group. (B) Remote time point. Percentage freezing during the context memory retention session for rats that underwent contextual fear conditioning 5 weeks prior to HPC surgery. Sham rats froze significantly more than either lesion group.

Figure 3 shows the percentage of time spent freezing during the retention test for the recent (A) and remote (B) time points. A two-way ANOVA with lesion condition as between-group factor (Sham, NMDA, and NMDA+TTX) and learning-surgery interval as between-groups factor (1 week and 5 weeks) revealed a significant main effect of lesion (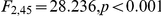) and no main effect of learning-surgery interval (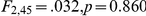). The lesion-interval interaction was not statistically significant (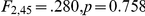) suggesting that treatment equally impaired both recent and remote memories. Analysis of the main effect of lesion revealed that HPC lesions caused retrograde amnesia. Specifically, pairwise comparisons of data collapsed across learning-surgery intervals indicated that the level of freezing by the Sham group was significantly greater than both NMDA and NMDA+TTX groups (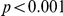). A significant difference was also found between freezing levels of the NMDA and NMDA+TTX groups (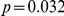).

NMDA mediated HPC damage significantly impaired both recent and remote contextual fear memory. When NMDA lesion-induced seizure activity was reduced, the severity of the memory impairment was also reduced, which suggests that the seizure activity contributed to the levels of retrograde amnesia observed in the NMDA alone groups. It is important to note that although the memory impairment was reduced, the NMDA+TTX groups still showed severe retrograde amnesia, and that this impairment was equivalent at both the recent and remote time points. Recent and remote contextual fear memories are equally susceptible to disruption by seizure activity, but this disruption in and of itself does not account for the full extent of retrograde amnesia observed following NMDA lesions.

## Discussion

The current experiments were designed to assess the effects of neurotoxic lesion induced seizure activity on the retrieval of recent and remote contextual fear memory. Seizure severity associated with NMDA lesions of the HPC was effectively limited by co-infusion with the sodium channel blocker TTX. We determined that NMDA induced HPC damage caused severe retrograde amnesia for contextual fear memory, regardless of the age of the memory. Importantly, this memory impairment was also severe for recent and remote memory when lesion-induced seizure severity was suppressed. Taken together, these experiments demonstrate that seizure activity following NMDA mediated HPC lesions is not completely responsible for the impairment of contextual fear memories, regardless of the age of the memory. We show that the HPC is necessary for the recall of recent as well as remote contextual fear memory, and confirm that the loss of HPC cells per se is driving the amnesia reported following HPC lesions in contextual fear tasks.

### Seizure activity can cause memory impairments

Seizure activity with a HPC focus is thought to have the potential to disrupt memories [5], [7]–[9]. The connection between seizure activity and amnesia has been well documented by research on humans [27]–[30], cats [31], and rodents [32]. In all of these cases, studies are presented showing either retrograde amnesia, anterograde amnesia, or both following periods of seizure activity. Studies in rats have assessed the relation between seizure activity and memory processes by manipulating the induction of seizure activity to affect the ability of a region such as the HPC or amygdala to consolidate or retrieve information, and this can be done pharmacologically [33], [34] or using electrical stimulation [24].

Given the known effects of seizure activity on learning and memory, it is reasonable to ask whether certain lesion techniques produce misleading results when assessing effects of loss of a specific neural structure on memory system function. NMDA, a potent excitatory amino acid, has severe effects on neurons by increasing the frequency of neuronal activity [35], creating sustained depolarization [35], and permitting excessive calcium influx from the extracellular space [36]–[38]. The neurotoxic effects of NMDA prompt an increase in osmolarity of the cell resulting in lysis [39]. Through these processes, many excitatory amino acids can have convulsant effects to varying degrees, of which NMDA has been found to produce the most pronounced seizure activity, that lasts for the longest duration [10]. We have shown that selective neurotoxic NMDA lesions of the HPC can lead to seizure activity (see Figure 2). Given that seizure activity can disrupt memory processes, and NMDA lesions cause seizure activity, these results leave open the possibility that NMDA induced seizure activity is responsible for retrograde amnesia found in certain studies of memory.

Consistent with other studies showing memory impairments following seizures, our current study suggests that seizure activity makes a modest contribution to retrograde amnesia following NMDA lesions. Indeed, when the groups at the recent and remote time points are collapsed, pairwise comparisons show that the NMDA alone group froze significantly less than the NMDA+TTX group. Although this effect is small when compared to the overall effect of HPC damage on freezing performance, decreasing seizure activity resulted in slightly less severe retrograde amnesia. Though a decrease in seizure activity resulted in better memory performance, lesioned rats were equally impaired at both the recent and remote time points. Because the contribution of seizure activity to the overall memory impairment is small for contextual fear conditioning, that is not to say that other tasks will be equally affected. Careful consideration should be given to this factor in all experimental designs that use neurotoxic lesions of the HPC.

### Equivalent retrograde amnesia for recent and remote contextual fear memories following complete NMDA lesions

The current findings are consistent with similar studies showing that lesions of the HPC made at a recent or remote time point after learning, produce robust retrograde amnesia for contextual fear memories [1], [2], [40], [41]. By manipulating the level of seizure activity produced by NMDA lesions, we have shown that the retrograde amnesia of rats in the high seizure versus low/no seizure groups is significant compared to the Sham control groups. This comparison is consistent across both recent and remote intervals. These results confirm that retrograde amnesia was due to removal of a memory system that contributes to the maintenance and expression of recent and remote contextual fear memories, and not due to disruptive seizure activity.

Equally robust retrograde amnesia for recent and remote memories is labeled a *flat gradient*, where the retrieval of the target memory is dependent on the integrity of the HPC. Our finding of a flat gradient following HPC damage adds support to the idea that the HPC plays a long-term, likely permanent, role in maintaining of contextual fear memory. This permanent role of the HPC in memory maintenance/retrieval is supported by other studies designed to directly assess the time-dependent role of the rat HPC in tasks such as learning to fear a discrete stimulus (tone or light) [2], [12]–[14], [42], spatial navigation [11], [43]–[47], object discrimination [1], [11], [44], [48], object exploration [49], shock-probe memory [50], and picture memory [51]. Though the pattern of flat gradients is predominant, a number of studies show a change in the dependence of retrieval on the HPC as the learning-to-lesion interval increases, where recent memories are more dependent on normal HPC functioning than remote memories–a term called *temporal gradient*. This change in HPC dependence has been reported in context fear conditioning [12]–[14], [17], trace eyeblink conditioning [52], trace fear [53], and flavour/odour memory [54]–[58]. The Standard Model of Systems Consolidation (SMSC) [21] accounts for temporal gradients by stating that certain memories that are dependent on the HPC, through a temporally based consolidation process become independent of the HPC. Though evidence supporting this theory in the rat is limited (see Sutherland et al. (2011, 2010) for comprehensive reviews [4], [15]), it stands as an essential part of the dominant view of HPC function in the rat literature.

One might predict that because of associated seizure activity, NMDA lesions would have greater disruptive effects on distal memory networks than other lesion techniques. If this was true, then memories contained in distal networks might be disrupted to a degree that, after HPC lesion, the original memories cannot be expressed. This level of disruption would make interpretation of retrograde amnesia following HPC damage difficult to attribute to the function of the HPC *per se*. Remote memories that are weakly established in non-HPC networks due to a systems consolidation process might be especially susceptible to disruptive seizure activity. If this is the case, and if context memories are consolidated outside the HPC with the passage of time, then the seizure activity produced during/after NMDA lesions could be responsible for the flat gradients observed in Table 1. Our present results count against this possibility by clearly showing that if levels of seizure activity are reduced during NMDA lesions, retrieval of both recent and remote contextual fear memory is equally impaired. Hence, NMDA induced seizure activity does not disrupt contextual fear memories that might be stored in non-HPC networks. The equivalent retrograde amnesia seen at both recent and remote time points adds direct support to the view that if contextual fear memories are initially dependent on the HPC, they will always require the HPC for retrieval–flat gradients should always be observed.

**Table 1:** Studies directly assessing temporally graded retrograde amnesia using contextual fear conditioning in rats.

Memory task	Damage	Lesion technique	RA duration^1^ (days)	Reference
Context fear	25% dorsal	Electrolytic	1 (27)	Kim and Fanselow (1992)
Context fear	25% dorsal	Electrolytic	1 (49)	Anagnostaras et al. (1999)
Context fear	40% dorsal	Neurotoxic (NMDA)	28 (71)	Maren et al. (1997)
Context fear	40% dorsal	Neurotoxic (NMDA)	Flat (180)	Lehmann, Lacanilao et al. (2007)
Context fear	85% d+v	Neurotoxic (NMDA)	Flat (180)	Lehmann, Lacanilao et al. (2007)
Context fear	40% dorsal	Neurotoxic (NMDA)	Flat (84)	Sutherland et al. (2008)
Context fear	40% ventral	Neurotoxic (NMDA)	Flat (84)	Sutherland et al. (2008)
Context fear	85% d+v	Neurotoxic (NMDA)	Flat (84)	Sutherland et al. (2008)
Context fear	70% d+v	Neurotoxic (NMDA)	1 (28)	Winocur et al. (2009)
Context fear	85% d+v	Neurotoxic (NMDA)	Flat (35)	Sparks et al. (current study)

1 RA duration is the number of days between learning and surgery at which there is significant RA. In brackets is a measure of uncertainty in the duration of RA indexed by the difference in days between the interval at which there is significant RA and the first remote interval when retention is significantly better.

Clearly, flat gradients have not always been observed using contextual fear conditioning in the rat. Maren et al. (1997) [14] used small NMDA lesions of the dorsal HPC, and reported that contextual fear memories 100 days old are less susceptible to HPC damage than recent memories (either 1 or 28 days old). At first glance, this result shows that with NMDA lesions a temporal gradient can be observed in this task. Though, after closer examination of the methods and data, the findings are not compelling and do not add strong support to the conclusions. The analysis in this paper requires more explanation in order to make explicit why the data may show temporally graded retrograde amnesia. For instance, the means for each group were calculated by selecting a single 64-s time bin (out of the 8 bins in the retention session) in which each independent rat showed the highest level of freezing. Using this method, the authors present a figure that shows a strong temporal gradient as the training-to-lesion interval increases. An approach as selective as this is not in line with the typical methodology of contextual fear analysis in the field. The typical method of quantifying freezing behaviour is to record freezing levels across a pre-defined extinction session and then average the levels of freezing found in each time bin (bin size being irrelevant) [1], [2], [12], [13]. Between-bin variability is not accounted for by selecting and reporting one bin from each rat.

Other evidence for temporally graded retrograde amnesia following HPC damage in context fear paradigms comes from studies using electrolytic lesions [12], [13]. As with the Maren et al. (1997) [14] study, these experiments used relatively small lesions of the HPC. As seen in Table 1, the two studies reporting a temporal gradient report damaged approximately 25% of the dorsal HPC [12], [13]. Studies in which HPC volume was quantitatively assessed following lesions show that spared tissue can support memory retrieval in tasks that involved picture discrimination memory and contextual fear memory [1], [2], [51]. The spared tissue in the studies by Kim & Fanselow (1992)[12] and Anagnostaras et al. (1999)[13] could have supported memory retrieval at the remote time points in their studies using electrolytic lesions. Because of the small amount of HPC tissue damaged in these studies, it is difficult to interpret the results as being strong support for the SMSC.

Of the experiments using large HPC lesions, a flat gradient is the norm [1], [2]. The one exception to this pattern is a study conducted by Winocur et al., (2009)[17], in which temporally graded retrograde amnesia was observed when HPC damage was performed either 1 or 28 days following training. One difference that sets this study apart from others using large lesions is that the rats were conditioned to a tone-shock pairing within the context. It is not clear how this procedural difference would lead to spared memories at a remote training-to-surgery interval. Using a range of small to large NMDA lesions, we have attempted to replicate studies showing temporally graded retrograde amnesia following tone-shock conditioning, and have consistently found flat gradients [2]. In further support of flat gradients, we have also investigated weak vs strong contextual fear conditioning [41], [59], small vs large lesions in dorsal, ventral, or dorsal+ventral HPC [2], and found significant retrograde amnesia at both recent and remote surgery-to-lesion time points. In all cases, no evidence of *temporally graded retrograde amnesia* have been found. On the contrary, *flat gradients* are the norm.

### Conclusion

Reduction of seizure activity produced during neurotoxic (NMDA) lesions of the HPC at a recent or remote time point did not spare contextual fear memories. Memories that were established 1 week or 5 weeks prior to surgery were equally susceptible to HPC damage. Together with the results from studies in rats using tasks such as fear to a discrete stimulus (tone or light) [2], [12]–[14], [42], spatial navigation [11], [43]–[47], object discrimination [1], [11], [44], [48], object exploration [49], shock-probe memory [50], and picture memory [51], the current study supports the supposition that if the HPC is involved in establishing a memory, it will always be involved in retrieval of that memory, either at a recent or remote time point. These results score against the view that the HPC plays a time limited role in the retrieval of certain types of memory, a view purported by the Standard Model of Systems Consolidation. Seizure activity produced by NMDA lesions of the HPC is unlikely to be responsible for the degree of retrograde amnesia for remote memories found in studies of the HPC and fear conditioning. A simpler view is supported here, that retrograde amnesia following HPC lesions is due specifically to the loss of cells within the HPC network.

## Methods

### Subjects

The University of Lethbridge Animal Care Committee approved all procedures under Protocol #0609, in accord with the guidelines set by the Canadian Council on Animal Care. Participants were 52 female Long-Evans rats (250–300 g) obtained from the Canadian Centre for Behavioural Neuroscience vivarium (University of Lethbridge, Alberta). Rats were housed in standard laboratory cages in a room with an ambient temperature of 21*°C*, 35% relative humidity, 12/12 hr light/dark cycle (lights on at 07∶00), and were provided with food and water *ad libitum*. Behavioural testing was conducted during the light phase of the daily cycle.

### Surgery

The rats were first anaesthetized with isoflurane (Janssen, Toronto, Ontario) in 1.0 L/min oxygen at 14.7 PSIA at 21°C (Benson Medical Industries, Markham, Ontario) and administered an analgesic (buprenorphine, 0.017 mg/kg, s.c.; Reckitt & Colman, Richmond, VA). They were then placed in a stereotaxic frame (Kopf instruments, Tujunga, CA) and a midline scalp incision was made and periosteum excised to expose the top of the skull. Small burr holes were drilled through the skull using the anterior/posterior and medial/lateral coordinates in Table 2. The HPC lesions were made by intra-HPC infusions of either *N*-methyl-D-aspartic acid (NMDA; 7.5 µg/µl in 0.9% saline; Sigma Chemical Co., St. Louis, MO) or NMDA co-infused with Tetrodotoxin citrate (TTX; 4 *ng/µ*l in 0.9% saline; Cedarlane Laboratories Ltd., Burlington, ON). The infusions were done sequentially through a 30-ga injection cannula attached to a 10 *µ*l Hamilton syringe via polyethylene tubing (PE-50). At the most ventral sites, a total volume of 0.5 *µ*l was infused at a flow rate of 0.15 *µ*l per minute. At the remaining 5 sites, a volume of 0.4 *µ*l was infused using the same flow rate. The injection needle was left in place for 3.5 min following the injection to facilitate diffusion. Following the lesions, the scalp incision was closed using sutures. As the rats recovered from the anaesthetic, a prophylaxis against seizures was administered (diazepam; 0.2cc; 10 mg/ml, i.p.; Sabex, Boucherville, Quebec). The same surgical procedures were used for the Sham rats except that no damage was done to the skull or brain. The rats were allowed to recover for a minimum of 10 days before subsequent conditioning or testing.

**Table 2:** Coordinates used for 7-site HPC lesion (measurements in millimetres relative to bregma).

Site	Anterior	Lateral	Ventral
1	–3.1	±1.5	–3.6
2	–4.1	±3.0	–4.0
3	–5.0	±3.0	–4.0
4	–5.0	±5.2	–7.3
5	–5.8	±4.4	–4.4
6	–5.8	±5.1	–7.5
7	–5.8	±5.1	–6.2

### Histology

After completion of the experiments, all animals were sacrificed by administering an overdose of sodium pentobarbital (100 mg/kg i.p.) and perfused transcardially with phosphate buffered saline (0.9% PBS) followed by 4% paraformaldehyde (PFA) in PBS. The brains were removed and post-fixed for 24 hr in PFA, then transferred and stored in 30% sucrose and PBS with sodium azide (0.02%) for at least 48 hr before sectioning. The brains were sectioned in the coronal plane 40 *µ*m thick using a cryostat microtome (-19C); every fourth section taken throughout HPC in all groups. Sections were wet-mounted on glass microscope slides and later stained with cresyl violet for visualization of HPC lesion induced damage and remaining tissue.

### Lesion Quantification

Volume of spared HPC tissue was calculated using the Cavalieri method. Images of cresyl violet stained sections from a single series (approximately 5 sections throughout the extent of the HPC) were taken using a Zeiss Axioskop 2 MotPlus epifluorescent scope attached to a QImaging Retiga CCD camera (Burnaby, British Columbia, Canada). Images were then analyzed using ImageJ software (http://rsb.info.nih.gob/ij/) in which a sampling grid with an area per point of 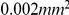 was created and randomly thrown over each image. The total number of points in contact with the HPC tissue in each section was counted. The number of points per section was multiplied by the area associated with each point, the section thickness and then the section sampling fraction. These numbers were then summed to provide the total estimated volume of the spared HPC tissue. Percent damage in each of the the lesioned rats was calculated by dividing the quantified spared tissue volume by the average HPC volume of the control group, then multiplying by 100.

### Apparatus and Procedures

#### Contextual Fear Conditioning

Conditioning and testing were carried out in four identical observation chambers (30×24×21 cm; MED-Associates, Burlington, VT). The chambers were constructed from aluminum (side walls) and Plexiglas (rear wall, ceiling, and hinged front door) and were situated in sound-attenuating cabinets located in a brightly lit and isolated room. The floor of each chamber consisted of 19 stainless steel rods (4 mm in diameter) spaced 1.5 cm apart (center to center). Rods were wired to a shock source and solid-state grid scrambler (MED-Associates) for the delivery of footshock unconditioned stimuli. The chambers were cleaned with dilute Quatsyl and stainless steel trays cleaned with the same solution were placed underneath the grid floors. Ventilation fans in each cabinet supplied background noise (65 dB, A scale).

For contextual fear conditioning procedures, rats were transported to the conditioning room four at a time in separate plastic transport tubs, placed in the conditioning chambers, and allowed to explore for 3 min before 5 foot shocks (2 s duration, 1 mA amplitude) were administered with an inter-shock interval of 58-s. The duration of the conditioning session for each rat was 8 min. Following the conditioning session, rats were immediately transported back to their home cage where they remained until retention testing. The retention session was conducted 11 days after surgery. Rats were transported to the conditioning room in the same manner as on the conditioning day; each animal was placed into the conditioning chamber for a 5 min extinction session. Behaviour while in the conditioning context was digitally recorded using FreezeFrame Video-Based Conditioned Fear System and analyzed by Actimetrics Software (Coulbourn Instruments, Wilmette, IL) for average freezing times. Freezing was defined as the absence of movement except for that due to respiration.

#### Evaluation of post-operative behavioural changes

The behaviour of each rat was continuously evaluated for 3 h following surgery, and observations were made according to a rating scale previously described by Sperk et al., 1985[25] and Baran et al., 1985[26] with slight modifications, developed by direct comparison with seizure scores used in the amygdala kindling model. The following scores were used for rating severity of NMDA induced seizures: 0, no seizures; 1, eye closure, twitching of vibrissae, sniffling, facial clonus, staring; 2, head nodding associated with more severe facial clonus; 3, unilateral or bilateral forelimb clonus; 4, rearing, often accompanied by bilateral forelimb clonus; 5, rearing with loss of balance and falling accompanied by generalized clonic seizures; 6, sustained generalized clonic convulsions (convulsive status epilepticus); 7, jumping/tonic seizure; and 8, respiratory arrest. The duration of each seizure score was recorded independently.

## References

[1] Lehmann H, Lacanilao S, Sutherland R (2007). Complete or partial hippocampal damage pro-duces equivalent retrograde amnesia for remote contextual fear memories.. European Journal of Neuroscience.

[2] Sutherland R, O'Brien J, Lehmann H (2008). Absence of systems consolidation of fear memories after dorsal, ventral, or complete hippocampal damage.. Hippocampus.

[3] Sutherland R, Lehmann H, Spanswick S, Sparks F, Melvin N (2006). Growth points in research on memory and hippocampus.. Canadian Journal of Experimental Psychology.

[4] Sutherland R, Sparks F, Lehmann H (2010). Hippocampus and retrograde amnesia in the rat model: a modest proposal for the situation of systems consolidation..

[5] Jarrard L, Meldrum B (1993). Selective excitotoxic pathology in the rat hippocampus.. Neuropathol-ogy and Applied Neurobiology.

[6] Albasser M, Poirier G, Warburton E, Aggleton J (2007). Hippocampal lesions halve immediate-early gene protein counts in retrosplenial cortex: distal dysfunctions in a spatial memory system.. European Journal o Neuroscience.

[7] McClelland J, McNaughton B, O'Reilly R (1995). Why there are complementary learning systems in the hippocampus and neocortex: insights from the successes and failures of connectionist models of learning and memory.. Psychological Review.

[8] Liang L, Ho Y, Patel M (2000). Mitochondrial superoxide production in kainate-induced hippocam-pal damage.. Neuroscience.

[9] Anagnostaras S, Gale G (2002). The hippocampus and pavlovian fear conditioning: Reply to bast et al.. Hippocampus.

[10] Zaczek R, Coyle J (1982). Excitatory amino acid analogues: neurotoxicity and seizures.. Neurophar-macology.

[11] Sutherland R, Weisend M, Mumby D, Astur R, Hanlon F (2001). Retrograde amnesia after hippocampal damage: Recent vs. remote memories in two tasks.. Hippocampus.

[12] Kim J, Fanselow M (1992). Modality-specific retrograde amnesia for fear.. Science.

[13] Anagnostaras S, Maren S, Fanselow M (1999). Temporally graded retrograde amnesia of contextual fear after hippocampal damage in rats: within-subjects examination.. The Journal of Neuroscience.

[14] Maren S, Aharonov G, Fanselow M (1997). Neurotoxic lesions of the dorsal hippocampus and pavlovian fear conditioning in rats.. Behavioural Brain Research.

[15] Sutherland R, Lehmann H (2011). Alternative conceptions of memory consolidation and the role of the hippocampus at the systems level in rodents.. Current Opinion in Neurobiology.

[16] Restivo L, Vetere G, Bontemi B, Ammassari-Teule M (2009). The formation of recent and remote memory is associated with time-dependent formation of dendritic spines in the hippocampus and anterior cingulate cortex.. The Journal of Neuroscience.

[17] Winocur G, Frankland P, Sekeres M, Fogel S, Moscovitch M (2009). Changes in context-specificity during memory reconsolidation: selective effects of hippocampal lesions.. Learning and Memory.

[18] Squire L, Alvarez P (1995). Retrograde amnesia and memory consolidation: a neurobiological per-spective.. Current Opinion in Neurobiology.

[19] Anagnostaras S, Gale G, FanselowM (2001). Hippocampus and contextual fear conditioning: Recent controversies and advances.. Hippocampus.

[20] Meeter M, Murre J (2004). Consolidation of long-term memory: evidence and alternatives.. Psy-chological Bulletin.

[21] Squire L, Stark C, Clark R (2004). The medial temporal lobe.. Annual Reviews in Neuroscience.

[22] Wiltgen B, Brown R, Talton L, Silva A (2004). New circuits for old memories: the role of the neocortex in consolidation.. Neuron.

[23] Frankland P, Bontempi B (2005). The organization of recent and remote memories.. Nature Reviews Neuroscience.

[24] Schmitz C, Hof P (2005). Design-based stereology in neuroscience.. Neuroscience.

[25] Sperk G, Lassmann H, Baran H, Seitelberger F, Hornykiewicz O (1985). Kainic acid-induced seizures: dose-relationship of behavioural, neurochemical and histopathological changes.. Brain Research.

[26] Baran H, Sperk G, Hortnagl H, Sapetschnig G, Hornykiewicz O (1985). α_2_-adrenoceptors modulate kainic acid-induced limbic seizures.. European Journal of Pharmacology.

[27] Butler C, Graham K, Hodges J, Kapur N, Wardlaw J (2007). The syndrome of transient epileptic amnesia.. Annals of Neurology.

[28] Butler C, Zeman A (2008). Recent insights into the impairments of memory in epilepsy: transient epileptic amnesia, accelerated long-term forgetting and remote memory impairment.. Brain.

[29] Hornberger M, Mohamed A, Miller L, Watson J, Thayer Z (2010). Focal retrograde amnesia: extending the clinical syndrome of transient epileptic amnesia.. Journal of Clinical Neuroscience.

[30] Milton F, Muhlert N, Pindus D, Butler C, Kapur N (2010). Remote memory deficits in transient epileptic amnesia.. Brain.

[31] Kesner R, Doty R (1968). Amnesia produced in cats by local seizure activity initiated from the amygdala.. Experimental Neurology.

[32] Sideroff S (1975). The relationship of seizures to retrograde amnesia in hippocampectomized rats.. Physiology & Behavior.

[33] Genkova-Papazova M, Lazarova-Bakarova M (1995). Pentylenetetrazole kindling impairs long-term memory in rats.. European Neuropsychopharmacology.

[34] Genkova-Papazova M, Shishkova B, Lazarova-Bakarova M (2001). Effect of the calcium channel blockers nifedipine and kiltiazem on pentylenetetrazole kindling-provoked amnesia in rats.. Euro-pean Neuropsychopharmacology.

[35] Olney J, McGeer E, Olney J, McGeer P (1978). Neurotoxicity of excitatory amino acids.. Kainic acid as a tool in neurobiology.

[36] Berdichevsky E, Riveros N, Sanchez-Armass S, Orrego F (1983). Kainate, n-methylaspartate and other excitatory amino acids increase calcium inux into rat brain cortex cells in vitro.. Neuroscience Letters.

[37] Griffiths T, Evans M, Meldrum B, Fuxe K, Roberts P, Schwarcz R (1983). Temporal lobe epilepsy, excitotoxins and the mechanism of selective neuronal loss..

[38] Retz K, Coyle J (1984). The differential effects of excitatory amino acids on uptake of ^45^
*cacl_2_* by slices from mouse striatum.. Neuropharmacology.

[39] Rothman S (1985). The neurotoxicity of excitatory amino acids is produced by passive chloride inux.. The Journal of Neuroscience.

[40] Lehmann H, Rourke B, Bernard J (2009). Single session contextual fear conditioning remains de-pendent on the hippocampus despite an increase in the number of context-shock pairings during learning..

[41] Sparks F, Spanswick S, Lehmann H, Sutherland R (2009). Neither time nor number of within-session context-shock pairings affect long-term dependence of memory on hippocampus..

[42] Lehmann H, Sparks F, O'Brien J, McDonald R, Sutherland R (2010). Retrograde amnesia for fear-potentiated startle in rats after complete, but not partial, hippocampal damage.. Neuroscience.

[43] Bolhuis J, Stewart C, Forrest E (1994). Retrograde amnesia and memory reactivation in rats with ibotenate lesions to the hippocampus or subiculum.. The Quarterly Journal of Experimental Psy-chology.

[44] Mumby D, Astur R, Weisend M, Sutherland R (1999). Retrograde amnesia and selective damage to the hippocampal formation: memory for places and object discriminations.. Behavioural Brain Research.

[45] Clark R, Broadbent N, Squire L (2005a). Hippocampus and remote spatial memory in rats.. Hip-pocampus.

[46] Clark R, Broadbent N, Squire L (2005b). Impaired remote spatial memory after hippocampal lesion despite extensive training beginning early in life.. Hippocampus.

[47] Martin S, de Hoz L, Morris R (2005). Retrograde amnesia: Neither partial nor complete hippocam-pal lesions in rats result in preferential sparing of remote spatial memory, even after reminding.. Neuropsychologia.

[48] Lehmann H, Glenn M, Mumby D (2007). Consolidation of object-discrimination memory is inde-pendent of the hippocampus in rats.. Experimental Brain Research.

[49] Gaskin S, Tremblay A, Mumby D (2003). Retrograde and anterograde object recognition in rats with hippocampal lesions.. Hippocampus.

[50] Lehmann H, Lecluse V, Houle A, Mumby D (2006). Retrograde amnesia following hippocampal lesions in the shock-probe conditioning test.. Hippocampus.

[51] Epp J, Keith J, Spanswick S, Stone J, Prusky G (2008). Retrograde amnesia for visual memories after hippocampal damage in rats.. Learning and Memory.

[52] Takehara K, Kawahara SS, Kirino Y (2003). Time-dependent reorganization of the brain compo-nents underlying memory retention in trace eyeblink conditioning.. Journal of Neuroscience.

[53] Quinn J, Ma Q, Tinsley M, Koch C, Fanselow M (2008). Inverse temporal contributions of the dorsal hippocampus and medial prefrontal cortex to the expression of long-term fear memories.. Learning and Memory.

[54] Winocur G (1990). Anterograde and retrograde amnesia in rats with dorsal hipocampal or dorso-medial thalamic lesions.. Behavioural Brain Research.

[55] Winocur G, McDonald R, Moscovitch M (2001). Anterograde and retrograde amnesia in rats with large hippocampal lesions.. Hippocampus.

[56] Clark R, Broadbent N, Zola S, Squire L (2002). Anterograde amnesia and temporally graded ret-rograde amnesia for a nonspatial memory task after lesions of hippocampus and subiculum.. The Journal of Neuroscience.

[57] Ross R, Eichenbaum H (2006). Dynamics of hippocampal and cortical activation during consolida-tion of a nonspatial memory.. The Journal of Neuroscience.

[58] Tse D, Langston R, Kakeyama M, Bethus I, Spooner P (2007). Schemas and memory con-solidation.. Science.

[59] Lehmann H, Sparks F, Spanswick S, Hadikin C, McDonald R (2009). Making memories independent of the hippocampus.. Learning and Memory.

